# Analysis of COVID-19 Transmission Sources in France by Self-Assessment Before and After the Partial Lockdown: Observational Study

**DOI:** 10.2196/26932

**Published:** 2021-05-04

**Authors:** Fabrice Denis, Anne-Lise Septans, Florian Le Goff, Stephan Jeanneau, François-Xavier Lescure

**Affiliations:** 1 Inter-regional Cancer Institut Jean Bernard - ELSAN Le Mans France; 2 Weprom Angers France; 3 Kelindi Lille France; 4 Adobis Group Grenoble France; 5 Infectious and Tropical Diseases Department Bichat-Claude Bernard University Hospital Assistance Publique – Hôpitaux de Paris Paris France

**Keywords:** COVID-19, web application, digital health, analysis, transmission, France, self-assessment, lockdown, observational, survey, impact, public health

## Abstract

**Background:**

We developed a questionnaire on a web application for analyzing COVID-19 contamination circumstances in France during the second wave of the pandemic.

**Objective:**

This study aims to analyze the impact on contamination characteristics before and after the second partial lockdown in France to adapt public health restrictions to further prevent pandemic surges.

**Methods:**

Between December 15 and 24, 2020, after a national media campaign, users of the sourcecovid.fr web application were asked questions about their own or a close relative’s COVID-19 contamination after August 15, 2020, in France. The data of the contamination’s circumstances were assessed and compared before and after the second partial lockdown, which occurred on October 25, 2020, during the second wave of the pandemic and was ongoing on December 24, 2020.

**Results:**

As of December 24, 2020, 441,000 connections on the web application were observed. A total of 2218 questionnaires were assessable for analysis. About 61.8% (n=1309) of the participants were sure of their contamination origin, and 38.2% (n=809) thought they knew it. The median age of users was 43.0 (IQR 32-56) years, and 50.7% (n=1073) were male. The median incubation time of the assessed cohort was 4.0 (IQR 3-5) days. Private areas (family’s or friend’s house) were the main source of contamination (1048/2090, 50.2%), followed by work colleagues (579/2090, 27.7%). The main time of day for the contamination was the evening (339/961, 35.3%) before the lockdown and was reduced to 18.2% (86/473) after the lockdown (*P*<.001). The person who transmitted the virus to the user before and after the lockdown was significantly different (*P*<.001): a friend (382/1317, 29% vs 109/773, 14.1%), a close relative (304/1317, 23.1% vs 253/773, 32.7%), or a work colleague (315/1317, 23.9% vs 264/773, 34.2%). The main location where the virus was transmitted to the users before and after the lockdown was significantly different too (*P*<.001): home (278/1305, 21.3% vs 194/760, 25.5%), work (293/1305, 22.5% vs 225/760, 29.6%), collective places (430/1305, 33% vs 114/760, 15%), and care centers (58/1305, 4.4% vs 74/760, 9.7%).

**Conclusions:**

Modalities of transmissions significantly changed before and after the second lockdown in France. The main sources of contamination remained the private areas and with work colleagues. Work became the main location of contamination after the lockdown, whereas contaminations in collective places were strongly reduced.

**Trial Registration:**

ClinicalTrials.gov NCT04670003; https://clinicaltrials.gov/ct2/show/NCT04670003

## Introduction

Patient-reported outcome applications have been shown to improve the health outcomes of patients including decreasing mortality [1-3].

We developed and launched a self-assessment and participatory surveillance web application called maladiecoronavirus.fr during the growing phase of the COVID-19 pandemic in March 2020 in France. This self-triage tool aimed to help patients who were symptomatic to be directed toward the emergency call or the general practitioner after analysis of symptoms and comorbidities. We showed that data from this web application could be a relevant tool to reduce the burden on emergency call centers [4]. It also proved to be useful in monitoring COVID-19 spread during the whole pandemic, with time and spatial correlations between number of hospitalizations and daily reported anosmia by users being higher than large-scale reverse transcription polymerase chain reaction (RT-PCR) positive tests [5,6]. A national partial lockdown was initiated in France on October 25, 2020, against the second wave of the COVID-19 pandemic. Contrary to the first complete lockdown from March to May 2020, this one maintained scholar, professional, and shopping activities. However, the circumstances of virus transmissions before and after this lockdown are not well known in France. We thus developed a specific questionnaire on a web application (sourcecovid.fr) for COVID-19 source of contamination analysis in France in December 2020 just after the second wave of the pandemic [7]. The objective of this national survey was to analyze the impact on contamination circumstances before and after the second partial lockdown in France initiated on October 25, 2020, associated with the second wave of the pandemic to optimize health public policy to further pandemic surges.

## Methods

Users of sourcecovid.fr were recruited via a national media campaign in France from December 15 to 25, 2020, including through social media, radio, and magazine campaigns, between December 15-18, 2020. Participants were recruited through the website. Respondents provided information on sociodemographic data, zip code, coexisting disorders anonymously, and the severity of their disease. Only symptomatic users were recruited. They were asked to enter data about their own contamination or the contamination of a close relative, about their sureness about the contamination’s circumstances (“I am sure,” “I think I know,” or “I don’t know”), and they also had to answer when, by who, and where they thought they (or the close relative) were contaminated. Users who answered “I don’t know” were excluded from analysis.

Questionnaires were excluded from the analysis if completion duration was considered inconsistent (below 100 or above 800 seconds); if users were asymptomatic; if they did not know about the contamination’s circumstances; and if contamination occurred before August 15, 2020, to reduce memory bias.

We excluded incubation times greater than 14 days from the analysis. Incubation time was calculated by comparing the date of presumed contamination and the date of first symptoms. The study was approved by the French National Health-Data Institute, which reviews ethical conduct of human participant research, data confidentiality, and safety. The website was not considered a medical device by regulatory authorities since no tracking was performed and data were anonymous. The web application did not have access to testing results. Access to the web application did not require a log-in or creating an account. The web application did not identify participants who responded several times.

Data of the contamination circumstances were assessed and compared before and after the second partial lockdown, which occurred on October 25, 2020, during the second wave of the pandemic and was ongoing on December 24, 2020. Fisher exact test was performed to assess changes in circumstances of contaminations.

## Results

As of December 24, 2020, 441,000 connections on the web application were observed. There were 2118 questionnaires assessable for analysis; 61.8% (n=1309) of the users were sure of their contamination circumstance, and 38.2% (n=809) thought they knew it. Sureness was not different according to age (*P*=.43). The median age of users was 43.0 (IQR 32-56) years, and 48.3% (1073/2218) were female. The total population older than 65 years made up 12.5% (n=265) of users, and 4.4% (n=93) of the questionnaires concerned people younger than 18 years. The median incubation time was assessable in 1676 questionnaires and was 4.0 (IQR 3-5) days. Whatever the sureness, time incubation was not different (*P*=.36). Among the incubation sample, 41.7% (699/1676) declared a positive RT-PCR or antigenic test.

Mild or moderate infection was reported by 85.1% (n=1802) of the 2118 questionnaires, severe infection in 10.8% (n=229), and hospitalization in 4.6% (n=98).

**Figure 1:**
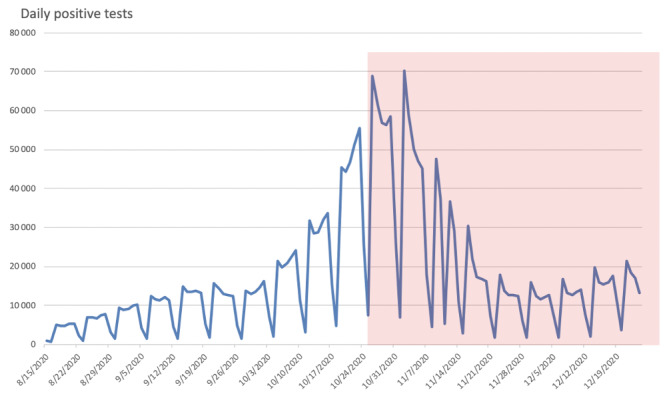
Impact of the partial lockdown on daily reverse transcription polymerase chain reaction positive tests. Red area: national partial lockdown period initiated on October 25, 2020.

The partial lockdown occurred on August 25, 2020, and was associated with an 80% reduction of daily contaminations (Figure 1).

During the period between August 15 and December 24, 2020, the private area (family and friends) was the main source of contamination in the 2090 questionnaires (n=1048, 50.2%) followed by work colleagues (n=579, 27.7%), or an unknown person (n=299, 14.3%), and 3.9% (n=83) did not know who contaminated them.

The lockdown occurred on October 25, 2020; 1334 questionnaires described the contamination’s circumstances between August 15 and October 24, 2020, and 784 described it between October 25 and December 24, 2020.

**Figure 2:**
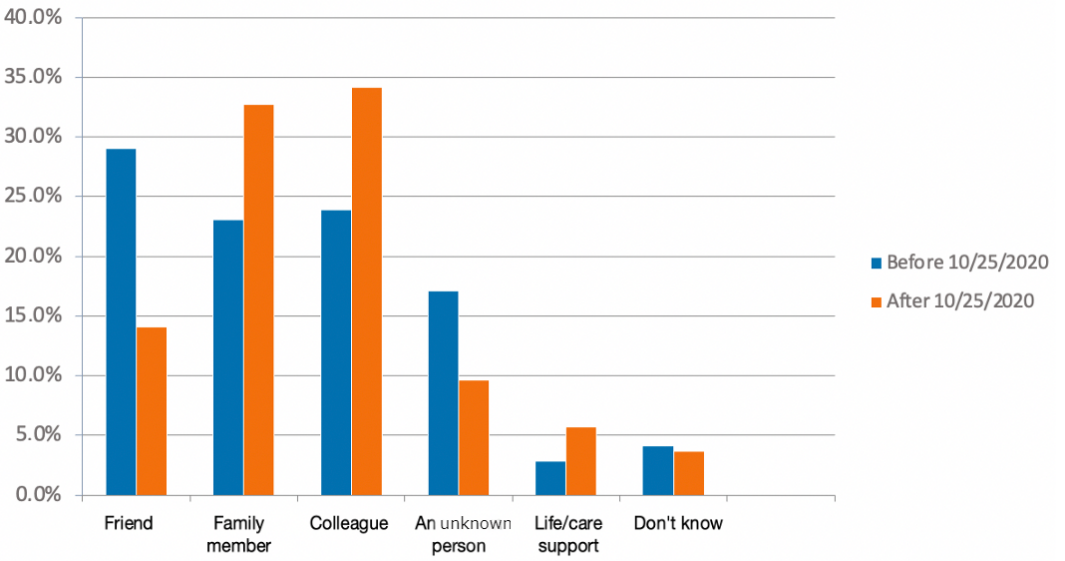
Contamination circumstances before versus after the partial lockdown, which was triggered in France on October 25, 2020. Answer to the question “Who contaminated you or your close relative?” (*P*<.001).

The person who transmitted the virus to the user before and after the lockdown was significantly different (*P*<.001): a friend (382/1317, 29% vs 109/773, 14.1%), a close relative (304/1317, 23.1% vs 253/773, 32.7%), and a work colleague (315/1317, 23.9% vs 264/773, 34.2%; *P*<.001; Figure 2).

The distribution of responses regarding the people who had contaminated the user was different between users who were sure and users who thought they knew the origin of their contamination (*P*<.001). If professional relation was privileged for both group, users were more, in proportion, likely to think it was an unknown person who infected them (151/790, 19.1% vs 148/1300, 11.4%).

**Figure 3:**
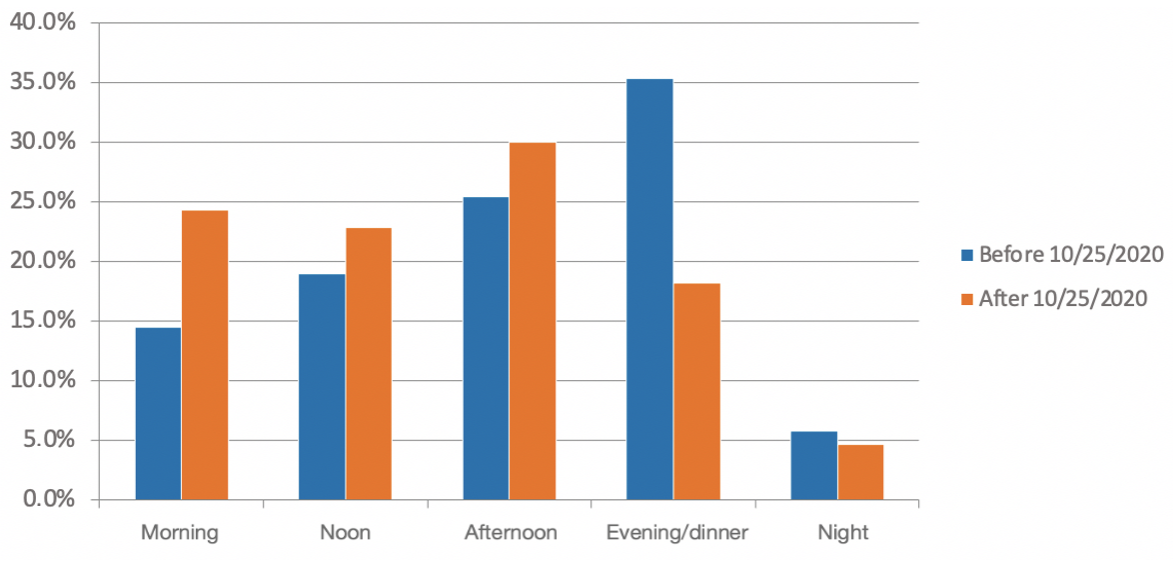
Contamination circumstances before and after the partial lockdown, which was triggered in France on October 25, 2020. Answer to the question “When do you think the contamination occurred?” (*P*<.001).

The main time of contamination also changed after the lockdown. Among people who knew the time of contamination (n=1434; n=961 before the lockdown; n=473 after the lockdown), the main time was the evening (339/961, 35.3%) before the lockdown, which was reduced to 18.2% (86/473) after the lockdown (*P*<.001). The main time of contamination became the morning after the lockdown initiation. Morning and noon together became 47.2% (223/473) of the contamination times (Figure 3).

The distribution of responses regarding the moment of contamination was different according to the level of certainty expressed by the user (*P*=.005). Even if the preferred time of day was the evening, it was observed that users who were less sure estimated more than users who were sure to have contracted the virus in the morning: 21.5% (114/529) vs 15.5% (140/905), respectively.

The distribution of responses regarding the location of suspected contamination was different according to the sureness of contamination (*P*=.003). For users who though they knew where or when they were contaminated, they privileged the collective place compared to users who were sure: 29.8% (231/775) vs 24.3% (313/1290), respectively.

**Figure 4:**
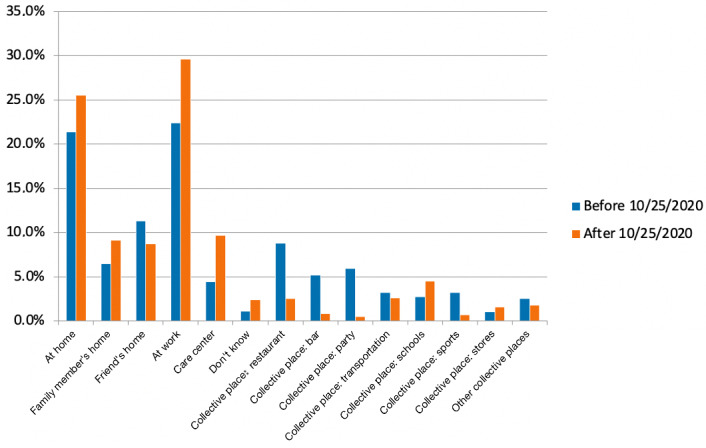
Contamination circumstances before and after the partial lockdown, which was triggered in France on October 25, 2020. Answer to the question “Where do you think the contamination occurred?” (*P*<.001).

The location of suspected contamination by users of the web application questionnaire was home (own home, family’s home, or friend’s home) in 39.1% (510/1305) of the declarations before the lockdown and remained high after the lockdown: 43.3% (329/760). The other locations where the virus was transmitted to the users changed before versus after the lockdown (*P*<.001). It was increased in the work area (293/1305, 22.5% before the lockdown vs 225/760, 29.6% after it), reduced in collective places (430/1305, 33.0% vs 114/760, 15.0%), and increased in care centers (58/1305, 4.4% vs 74/760, 9.7%). Work became the main location of contamination after the lockdown (Figure 4).

**Figure 5:**
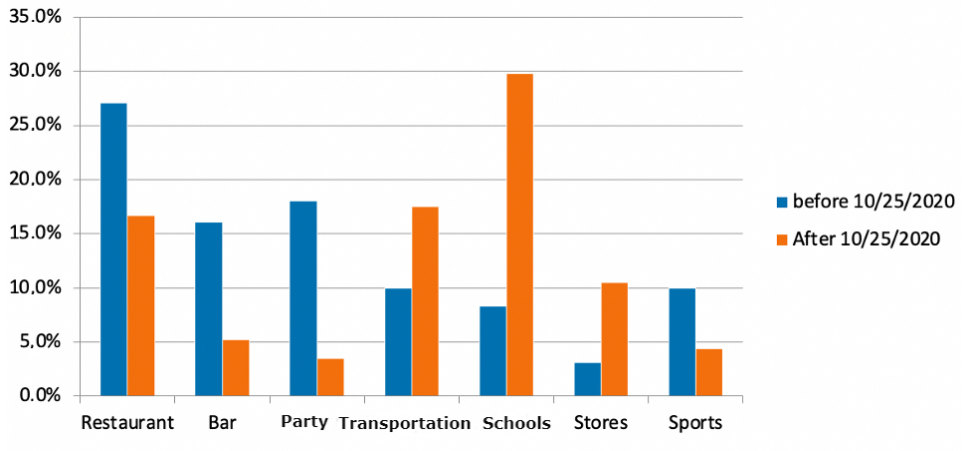
Main collective places concerned by contaminations before and after lockdown (*P*<.001); only principal location of collective area).

Collective places where transmission occurred were significantly different before and after the lockdown (*P*<.001). The main collective places in terms of virus transmission before the lockdown were restaurants with a reduction from 27.0% (114/422) to 16.7% (19/114), bars (from 68/422, 16.1% to 6/114, 5.2%), parties (from 76/422, 18.0% to 4/114, 3.5%), and sports (from 42/422, 10.0% to 5/114, 4.4%). Among collective places, transportation and schools became the main collective places of transmissions after the partial lockdown: 17.5% (20/114) and 29.8% (34/114), respectively (Figure 5).

Among collective places, other collective places than those previously cited were reported by 4.5% (19/422) and 11.4% (13/114) of the users before and after the lockdown initiations, respectively (19/1334, 1.5% and 13/784, 1.7% of all contaminations, respectively).

For the total contaminations, restaurants were the source of only 2.5% (19/760) of infections after the lockdown versus 8.8% (114/1297) before the lockdown; bars were the source for 0.8% (6/760) of infections versus 5.2% (68/1297) before the lockdown; schools and stores were the source of 4.5% (34/760 vs 35/1297, 2.7%) and 1.6% (12/760 vs 13/1297, 1.0%, respectively) before the lockdown.

## Discussion

Our results suggest that virus transmission occurred mainly in private areas (1048/2090, 50.2%) between August 15 and December 24, 2020, in France or by a colleague at work (579/2090, 27.7%). The partial lockdown, which occurred on October 25, 2020, during the second wave of COVID-19, changed the circumstances of contamination.

Contamination by a friend was significantly reduced (382/773, 29.0%) before and (109/773, 14.1%) after the lockdown but increased from a family member or a colleague. Among people who knew the time of contamination, the main time of the day was the evening before the lockdown (339/961, 35.3%), and the morning and noon became the main time of contamination after the lockdown (86/473, 18.2%). The main location of suspected contamination by users of the web application questionnaire was private home (own home, family’s home, or friend’s home) in 39.1% (510/1305) of declarations before the lockdown and remained high after the lockdown (329/760, 43.3%).

Contaminations increased in the work area (293/1305, 22.5% vs 225/790, 29.6%; *P*<.001), reduced in collective places (430/1305, 33% vs 114/760, 15.0%; *P*<.001), and increased in care centers (58/1305, 4.4% vs 74/760, 9.7%; *P*<.001). Work became the main location of contamination after the lockdown. The main collective places impacted by the shutdown were restaurants, bars, and parties in which reduction of contaminations was significant.

Our results showed that contaminations occurred in collective places 33.0% (430/1305) of the time, and that the lockdown reduced it to 15.0% (114/760) of contaminations (ie, a 54.5% reduction), whereas daily anosmia reported on the national website maladiecoronavirus.fr showed an 80% reduction after the partial lockdown initiation. This can be explained by the voluntary decisions of people to reduce social meetings at a higher rate than government-imposed restrictions on activity during the lockdown and especially among friends, from which contaminations decreased from 29.0% to 14.1% [8]. As work and school were maintained during the shutdown, higher rates of contaminations were observed in those places. The partial lockdown concerned all cultural locations such as theaters or cinemas. We did not ask users of the web application sourcecovid.fr specifically if they thought they were contaminated in those sites but only “other locations.” However, “other locations” was answered by nearly 1.5% of users before and after the lockdown, suggesting that cultural sites were not a significant source of contamination.

Although this study is based on unverifiable data, the quality of the data is consolidated by the incubation period calculated at 4.0 days, which is the median time reported in the literature; the equity between males and females; 10.8% (229/2118) of users with severe disease (15% in Guan et al [9]); the median age of users (43 years in our study, 47 years in Guan et al [9]); and 61.8% (1309/2118) of users reporting sureness of contamination circumstances. Moreover, results were consistent with published reports on excess of contamination risk in restaurants (times 2.4) or bars (times 3.9) and households (times 10), with a higher number of questionnaires in our study (n=2218) than in Fisher et al [10-12].

Our study has limitations. A user of the web application could use it several times and could have filled out more than one questionnaire. Memory bias could occur to users contaminated at the beginning of the study period, which explains the relative high number of users during the recent lockdown period.

The population older than 65 years made up 12.5% (265/2118) of users, whereas contamination circumstances were not the same as in the active population. Moreover, only 4.4% (93/2118) of questionnaires concerned people younger than 18 years. This has probably underestimated contaminations at school. We do not have enough data to assess geographic variations of the lockdown effect, especially to differentiate urban and rural area impact.

However, our study is the first to assess a partial lockdown effect on contamination circumstances and may help health authorities to adapt a policy of preventing COVID-19 spread.

## Abbreviations

RT-PCR: reverse transcription polymerase chain reaction
